# Effects of glutamine on plasma protein and inflammation in postoperative patients with colorectal cancer: a meta-analysis of randomized controlled trials

**DOI:** 10.1007/s00384-023-04504-8

**Published:** 2023-08-11

**Authors:** Kai Xiong, Guangsong Li, Yu Zhang, Tiantian Bao, Ping Li, Xiangdong Yang, Jiang Chen

**Affiliations:** 1 College of Clinical Medicine, Guizhou University of Traditional Chinese Medicine, Guiyang, No. 50 Shi East Road, Nanming District Guiyang, 550002 China; 2 Department of Pharmacy, the First Affiliated Hospital of Guizhou University of Traditional Chinese Medicine, Guiyang, 550002 China; 3 Colorectal and Anal Surgery, Chengdu Anorectal Hospital, Chengdu, 610015 China; 4 Colorectal and Anal Surgery, the First Affiliated Hospital of Guizhou University of Traditional Chinese Medicine, Guiyang, 550002 China

**Keywords:** Colorectal cancer, Glutamine, Nutritional status, Inflammation, Meta-analysis

## Abstract

**Objective:**

To evaluate the effects of glutamine on the plasma protein and inflammatory responses in colorectal cancer (CRC) patients following radical surgery.

**Methods:**

We thoroughly retrieved online databases (EMBASE, MEDLINE, PubMed, and others) and selected the randomized controlled trials (RCTs) with glutamine vs. conventional nutrition or blank treatment up until March 2023. The plasma protein associated markers indicators (consisting of albumin (ALB), prealbumin (PA), nitrogen balance (NB), total protein (TP)), inflammatory indicators (including TNF-α, CRP, infectious complications (ICs)), and matching 95% confidence intervals (CIs) were evaluated utilizing the pooled analysis. Subsequently, meta-regression analysis, contour-enhanced funnel plot, Egger’s test, and sensitivity analysis were carried out.

**Results:**

We discovered 26 RCTs, included an aggregate of 1678 patients, out of which 844 were classified into the glutamine group whereas 834 were classified into the control group. The findings recorded from pooled analysis illustrated that glutamine substantially enhanced the plasma protein markers (ALB [SMD_[random-effect]_ = 0.79, 95% CI: 0.55 to 1.03, *I*^2^ = 79.4%], PA [SMD_[random-effect]_ = 0.94, 95% CI: 0.69 to 1.20, *I*^2^ = 75.1%], NB [SMD_[random-effect]_ = 1.11, 95% CI: 0.46 to 1.75,* I*^2^ = 86.9%). However, the content of TP was subjected to comparison across the 2 groups, and no statistical significance was found (SMD_[random-effect]_ = − 0.02, 95% CI: − 0.60 to 0.57,* P* = 0.959, *I*^2^ = 89.7%). Meanwhile, the inflammatory indicators (including TNF-α [SMD_[random-effect]_ = − 1.86, 95% CI: − 2.21 to − 1.59, *I*^2^ = 56.7%], CRP [SMD_[random-effect]_ = − 1.94, 95% CI: − 2.41 to − 1.48, *I*^2^ = 79.9%], ICs [RR_[fixed-effect]_ = 0.31, 95% CI: 0.21 to 0.46, *I*^2^ = 0.00%]) were decreased significantly followed by the treatment of glutamine.

**Conclusions:**

The current study’s findings illustrated that glutamine was an effective pharmaco-nutrient agent in treating CRC patients following a radical surgical operation.

PROSPERO registration number: CRD42021243327.

**Supplementary Information:**

The online version contains supplementary material available at 10.1007/s00384-023-04504-8.

## Introduction

Colorectal cancer (CRC) is a common malignancy that most predominantly affects the gastrointestinal tract and has become a serious health concern. Data worldwide illustrated that 1,880,725 newly increased CRC patients in 2020, and the CRC mortality rate was expected to be about 9.4% [1]. In addition, in 2035, the death rates of colon and rectal cancers are expected to rise by 60% and 71.5%, correspondingly [2]. Meanwhile, the incidence and mortality rate was still rising in people younger than 50 years by year [3].

Radical surgery is the most effective and irreplaceable treatment for nonmetastatic or resectable CRC in the clinic [4]. Nevertheless, owing to the high-level tumor consumption prior to the CRC radical resection, inadequate nutritive intake, and the stress responsiveness associated with postoperative trauma, CRC patients have a great likelihood of suffering from postoperative complications, inflammatory injury, intestinal dysfunction, and malnutrition (lower level of plasma protein and negative nitrogen balance) [5, 6]. It is well known that the level of plasma protein is one of the critical parameters of the assessment of nutritional status in clinical practice. Malnutrition incidence has been observed to attain 15–40% among cancer patients at the time of diagnosis, and up to 80–90% among advanced cancer patients, according to previous study [7]. The incidence is also common in CRC patients, ranging from 45 to 60%, and such prevalence rate rises dramatically following the radical surgical intervention [8]. Thus, CRC patients have a decreasing rate of overall survival (OS) and recurrence-free survival after colectomy due to sustained malnutrition or worsening nutritional status [9]. In addition, inflammation is recognized as a hallmark of tumors that substantially contributes to cancer development, progression, and recrudescence [10]. Systemic inflammation could be initiated or aggravated after curative resection in CRC patients. There was increasing evidence that systemic inflammation promoted the aggressive behavior and progression of tumors and even significantly shortened the survival time of cancer patients [11]. Many studies have attributed worse prognosis, tumor progression, invasion, and angiogenesis to systemic or local inflammation induced by radical surgery [11–13].

Glutamine, an integral constituent of immunological nutrition, is an important source of energy for the digestive system and may help to enhance nutritional status [14, 15]. In general, animals and humans are rarely deficient in glutamine, but glutamine becomes an “essential amino acid” due to the significant decrease in serum glutamine concentration in patients with tumors or severe traumatic stress [14]. The levels of glutamine levels might remarkably affect the time of progression-free survival (PFS) and OS, and might be utilized as a prognostic marker of CRC patients [16, 17]. Many studies have reported that glutamine and glutamine-containing dipeptides used in CRC patients reduced infection-related complications, pneumonia, duration of hospitalization, and improved intestinal permeability and absorption [18, 19]. However, in previous clinical trials, glutamine supplementation did not substantially reduce the incidence of postoperative complications or enhance survival outcomes among CRC patients [20, 21]. Moreover, a randomized controlled trial by Ma et al. demonstrated that glutamine-rich EN did not play an immunomodulatory role compared to standard EN [22]. Furthermore, there is still no specific guideline for the application of glutamine in terms of time and amount of supplementation for patients with CRC, even the ESPEN guidelines [7].

Owing to the differences in research design, demographic selection, sample sizes, and systematic methodologies used in current clinical investigations, all available evidence was difficult to undergo comparison. Therefore, we applied a meta-analysis for RCTs on glutamine used in CRC patients who received radical resection to clarify such misunderstandings and assess glutamine’s therapeutic value in CRC patients.

## Materials and methods

### Protocol registration

This meta-analysis protocol was registered earlier in April 2021 in PROSPERO (number: CRD42021244182, https://www.crd.york.ac.uk/PROSPERO).

### Patient and public involvement

Patients and/or the public were not involved.

### Eligibility criteria

This study referred to the PRISMA 2020 Checklist (Supplement 1) and the Cochrane Handbook for Systematic Reviews of Interventions. This research’s inclusion and exclusion criteria referred to the “PICOS” principles. The eligibility guidelines below were used to include the studies: (1) RCTs were used in designing the original studies; (2) patients who have had a curative resection for CRC (both rectal and colon cancers); (3) the experimental cohort received glutamine, whereas the control cohort received either normal nutrition or blank treatment (fluid supportive therapy); (4) original RCTs reported a minimum of one of the examined outcomes. The following factors were used to exclude studies that were not eligible: (1) CRC patients with severe immune system disease or metabolic syndrome; (2) studies that are not relevant and materials that are duplicated; (3) data of outcomes not accessible in the literature; (4) reviews, case reports, comments, letters, laboratory studies, and meta-analysis.

### Search strategy

Up until March 2023, we thoroughly screened online databases, which included Wanfang, VIP medicine information system, Chinese Biomedical Database, China National Knowledge Infrastructure (CNKI), Cochrane Library, Embase, MEDLINE, and PubMed. Medical subject headings (MeSH) phrases were combined with the free words below in the search queries: (Colorectal / Rectal / Colon / cancer / tumor / carcinoma / neoplasm) AND (glutamine / Gln/ nutrition / immune-nutrition) AND (albumins / albumin / prealbumin / protein/ nitrogen) AND (inflammation / infection / TNF-α / CRP) AND (random / randomized / RCTs / clinical trial / trials). The detailed syntax for PubMed database was provided as Supplement 2. Furthermore, possible references were manually collected. The language of the retrieved studies was limited to Chinese or English.

### Study selection

The software of Endnote™, Version X8 (Thompson Reuters) was used to integrate all of the search outcomes. Manual removal of duplicates was performed. The initial screening of original articles was conducted separately by two researchers (Kai Xiong and Yu Zhang). The researchers reviewed the whole text of the literature to assess whether the study meets the eligibility requirements. Disagreements were resolved by dialogue or judged by a third party.

### Extraction and analysis of data

By utilizing a standardized form, all data were obtained separately from the primary RCTs by two researchers (Guangsong Li and Kai Xiong). The details below were taken from the data: (1) study ID, which includes the first author’s name and the year of paper was published; (2) study participants (male and female), sample sizes, and their ages; (3) the treatment and control subgroups’ therapy regimens, including the intervention time, dosage of drugs and duration of intervention; (4) the primary endpoint—plasma protein indicators (encompassing albumin (ALB), prealbumin (PA), nitrogen balance (NB), total protein (TP)) and the secondary endpoint—inflammatory indicators (including TNF-α, CRP, and infectious complications (ICs)) were reported in original studies when the treatment ends. If we identified inadequate data, we contacted the authors for more details. Dialogue and agreement were used to resolve differences between the two researchers.

### Assessment of quality

The GRADE of Cochrane Collaboration Network was applied to assess the grades of evidence for this study. Regarding quality assessment, the Cochrane Collaboration’s technique for evaluating the risk of bias was utilized. Iteration, debate, and consensus were used to address any issues that arose during the evaluation.

### Statistical analysis

Stata version 14.0 (Stata Corporation) was utilized to conduct analyses of all statistical data. Prior to calculating the pooled effect, the heterogeneity of the studies was examined utilizing an *I*^2^ test a *Q* test. The *I*^2^ and *Q* tests findings were used to determine whether a random or fixed effects model should be used. If *I*^2^ was less than 50% and P was greater than 0.1, a fixed-effects model was utilized. If not, a random-effect model was utilized. The variables ALB, PA, TP, NB, TNF-, and CRP had a continuous endpoint, and the pooled analyses of these markers were reported as standardized mean difference (SMD). The IC was a dichotomous variable, and the results of pooled analyses were reported as relative risks (RR). A *Z* test was utilized to measure the significance of pooled effects; *P* < 0.05 was established as the criterion for statistical significance. The impact of high-risk research on the entire meta-analysis was investigated utilizing sensitivity analysis. To identify possible publication bias and the specific reasons causing publication bias, the contour-enhanced funnel plot and Egger’s regression asymmetry test were utilized. Meta-regression conducted via a random-effect model was utilized to investigate the likely cause of heterogeneity. In meta-regression, the constrained maximum likelihood estimation approach developed by Harbord et al. [23] was employed.

## Results

### Result of study selection

**Fig. 1:**
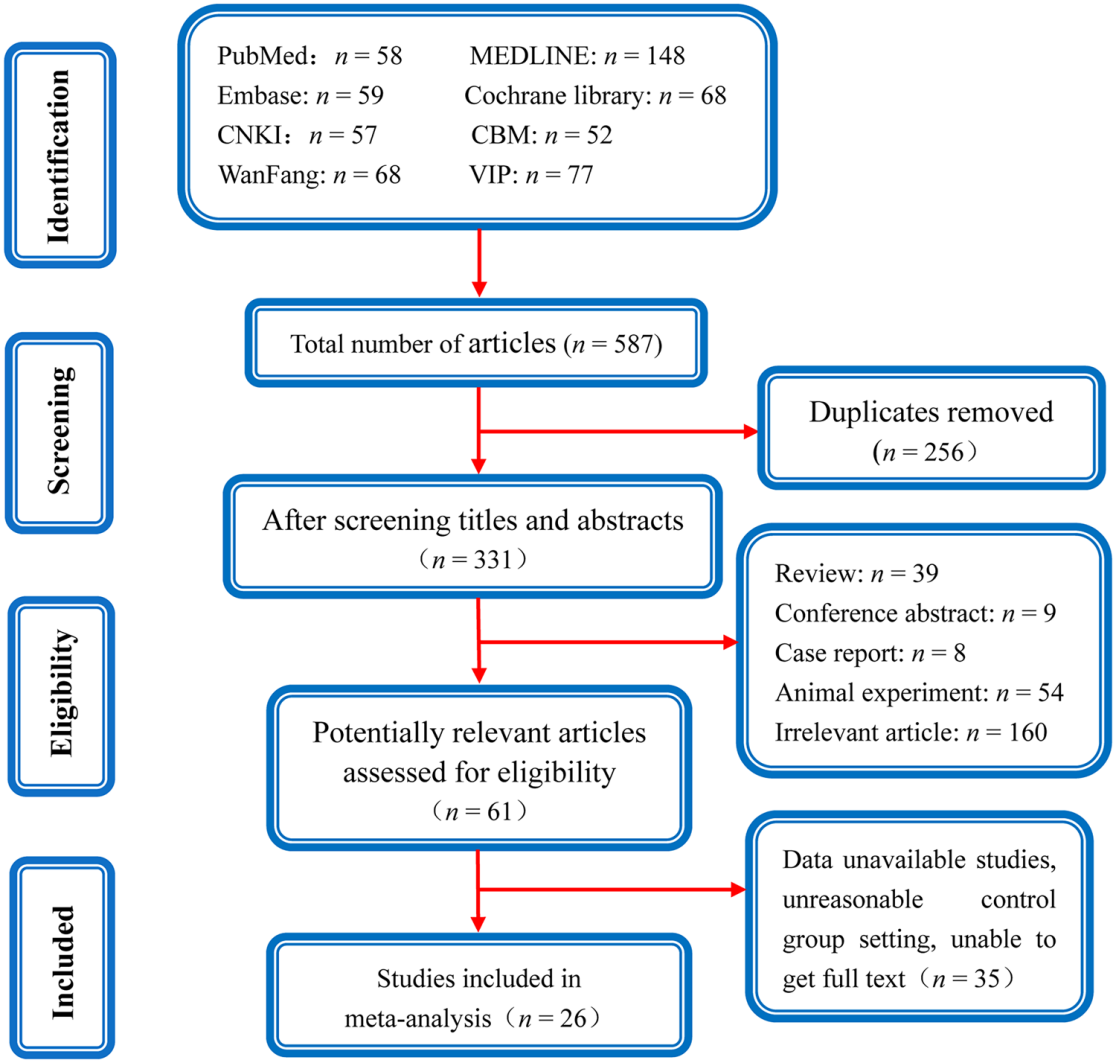
Flowchart depicting the screening procedure

In the end, 587 related articles were acquired from the databases. Two hundred fifty-six of them were records that had previously been reported. The remaining publications were then filtered for conference abstracts, animal studies, reviews, and case reports, which resulted in the exclusion of 270 articles, leaving only 61 studies. By reviewing the abstracts or full texts, 35 papers that did not meet the eligibility criteria were excluded. In the end, this meta-analysis included 26 RCTs that fulfilled the required criteria. The article selection procedure is depicted in Fig. 1.

### Study features

In total, 1678 participants was enrolled in 26 different studies [24–49]. Among these, 844 patients were classified into the glutamine cohort, whereas 834 were classified into the control cohort. Table 1 shows the key features of the 26 RCTs that were selected. The chosen 26 articles were published between the years 1994 to 2021. Glutamine was administered via enteral nutrition (EN) in 8 of the RCTs [24–31] and via parenteral nutrition (PN) in eighteen RCTs [32–49]. In terms of results for nutrition-related indicators, 22 RCTs [24–31, 33–41, 43–45, 47, 48] reported ALB content, while 16 RCTs [26, 27, 29–31, 34, 35, 37, 38, 40, 41, 43–45, 47, 48] reported PA content, 8 studies [24–26, 28, 37, 40, 45, 48] demonstrated TP content, and 7 trials [31, 33, 35, 36, 39, 41, 43] reported NB indicator. Additionally, the results of inflammatory indicators, including TNF-α content, was described in 10 RCTs [27, 29, 32, 40, 42, 44–47, 49], CRP content, was reported by 6 trials [29, 30, 40, 45, 47, 49], and the ICs (such as incision infection, lung infection, abdominal infection) was reported by 11 trials [27, 30, 31, 33, 34, 36, 38, 41, 44, 46, 49]. Table 1 shows the details of the articles that were included.

**Table 1:** The major data from the selected studies in the meta-analysis

Study ID	Sample size (*n*)		Ages (year)		Tumor types	Dose of glutamine	Route of administration	Region	Outcomes
	Control	Treatment	Control	Treatment					
O'Riordain et al. [32]	10 (M/F: 5/5)	10 (M/F: 7/3)	69 (58–74)	65 (59–78)	CRC (CC = 7, RC = 13)	AOD_1_-_6_: 0.18 g/(kg·day)	PN	UK	⑤
Li et al. [33]	20 (M/F: 10/10)	20 (M/F: 11/9)	58.02 ± 4.63	57.81 ± 3.75	CRC (CC = 19, RC = 21)	AOD_1_-_7_: 0.2 g/(kg·day)	PN	China	①③⑦
Chen and Lin [34]	24 (M/F: 17/7)	24 (M/F: 15/9)	68.12 ± 4.46	66.84 ± 5.52	CC	POD_1-5_-AOD_1-7_: 0.4 g/(kg·day)	PN	China	①②⑦
Cao and Zhou [35]	15 (M/F: NR)	15 (M/F: NR)	NR		CRC (CC = NR, RC = NR)	POD_1-5_-AOD_1-5_: 0.2 g/( kg·day)	PN	China	①②③
Xu et al. [36]	30 (M/F: NR)	30 (M/F: NR)	NR		CRC (CC = NR, RC = NR)	AOD_1-7_: 0. 2 g/(kg·day)	PN	China	①③⑦
Chen et al. [37]	22 (M/F: 14/8)	25 (M/F: 15/10)	49.7 ± 7.0	51.2 ± 7.2	CRC (CC = 35, RC = 12)	AOD_2-10_: 0.35 g/(kg·day)	PN	China	①②④
Yang and Li [38]	20 (M/F: 10/10)	24 (M/F: 13/11)	61.1	60.2	CC	AOD_1-7_: 100 ml/day	PN	China	①②⑦
Yuan et al. [39]	30 (M/F: 15/15)	30 (M/F: 15/15)	61.83 ± 6.23	62.57 ± 7.38	CRC (CC = 26, RC = 34)	POD_1-4_-AOD_1-5_: 0.4 g/(kg·day)	PN	China	①③
Jiang et al. [40]	31 (M/F: 16/15)	29 (M/F: 18/13)	58.2 ± 9.5	56.8 ± 10.2	CRC (CC = NR, RC = NR)	POD_1-3_-AOD_1-7_: 0.4 g/(kg·day)	PN	China	①②④⑤⑥
Zhang [24]	20 (M/F: NR)	20 (M/F: NR)	NR		CRC (CC = NR, RC = NR)	AOD_1-7_: 30 g/day	EN	China	①④
Bu et al. [41]	24 (M/F:13/11)	24 (M/F: 11/13)	66. 8 ± 10.9	70.5 ± 10.6	CRC (CC = 23, RC = 25)	AOD_1-7_: 0.5 g/(kg·day)	PN	China	①②③⑦
Cui [42]	20 (M/F:10/10)	20 (M/F: 12/8)	59 ± 11.9	55 ± 10.8	CC	POD_1_-AOD_1_: 0.5 g/(kg·day)	PN	China	⑤
Chen [25]	22 (M/F: NR)	22 (M/F: NR)	58.7 ± 6.7		CRC (CC = NR, RC = NR)	AOD_1-10_: 30 g/day	EN	China	①④
Luo et al. [43]	23 (M/F: 12/11)	23 (M/F: 10/13)	51.9 ± 2.6	53.2 ± 3.8	CC	POD_1-3_-AOD_1-7_: 0.5 g/(kg·day)	PN	China	①②③
Cheng and Huang [44]	50 (M/F: 26/24)	50 (M/F: 28/22)	NR		CC	POD_1-3_-AOD_1-7_: 100 ml/day	PN	China	①②⑤⑦
Li and Li [26]	30 (M/F: 18/12)	30 (M/F: 18/12)	50.5 ± 4.9	50.1 ± 4.6	RC	AOD_1-7_: 0.4 g/(kg·day)	EN	China	①②④
Liu et al. [45]	42 (M/F: 25/17)	43 (M/F: 24/19)	58.2 ± 10.1	57.1 ± 9.8	CRC (CC = 25, RC = 60)	POD_1-3_-AOD_1-7_: 0.4 g/(kg·day)	PN	China	①②④⑤⑥
Zheng [46]	55 (M/F: NR)	55 (M/F: NR)	NR		CC	POD_1-3_-AOD_1-7_: 100 ml/day	PN	China	⑤⑦
Huang et al. [47]	63 (M/F: 31/32)	63 (M/F: 33/30)	35–67	32–69	CC	POD_1-3_-AOD_1-7_: 100 ml/day	PN	China	①②⑤⑥
Liu et al. [27]	40 (M/F: 25/15)	40 (M/F: 21/19)	59.1 ± 7.5	61.4 ± 7.0	CC	AOD_1-7_: 100 ml/day	EN	China	①②⑤⑦
Chen et al. [48]	42 (M/F: 24/18)	42 (M/F: 22/20)	62.7 ± 11. 3	62.1 ± 10.6	CC	AOD_1-7_: 0. 5 g/(kg·day)	PN	China	①②④
Li and Jia [28]	32 (M/F: 19/13)	32 (M/F: 19/13)	65.5 ± 9.0	62.6 ± 9.6	CRC (CC = 24, RC = 40)	AOD_1-7_: 0.5 g/(kg·day)	EN	China	①④
Zhao [49]	28 (M/F: 19/9)	32 (M/F: 22/10)	54.42 ± 5.21	56.75 ± 5.60	CRC (CC = 32, RC = 28)	POD_1-3_-AOD_1-7_: 50 ml/day	PN	China	⑤⑥⑦
De et al. [29]	52 (M/F: 29/23)	52 (M/F: 27/25)	53.24 ± 11.38	53.54 ± 11.57	CC	AOD_1-7_: 100 ml/day	EN	China	①②⑤⑥
Cai et al. [30]	56 (M/F: 34/22)	56 (M/F: 31/25)	68.3 ± 3.6	67.2 ± 3.5	RC	AOD _2–3_: 5 g/d, AOD _4–7_: 10 g/day	EN	China	①②⑥⑦
Li et al. [31]	33 (M/F:18/15)	33 (M/F: 19/14)	60.39 ± 7.02	± 7.05	CRC (CC = 43, RC = 23)	AOD_1-7_: 5 g/(kg·day)	EN	China	%1 ②③⑦

①: Albumin (ALB); ②: prealbumin (PA); ③: nitrogen balance (NB); ④: total protein (TP); ⑤: tumor necrosis factor-alpha (TNF-α); ⑥: C-reactive protein (CRP); ⑦: infectious complications (ICs)

*NR* not report, *POD* pre-operation day, *AOD* after-operation day, *M* male, *F* female, *CRC* colorectal cancer, *RC* rectal cancer, *CC* colon cancer, *EN* enteral nutrition, *PN* parenteral nutrition

### GRADE system evaluation

**Fig. 2:**
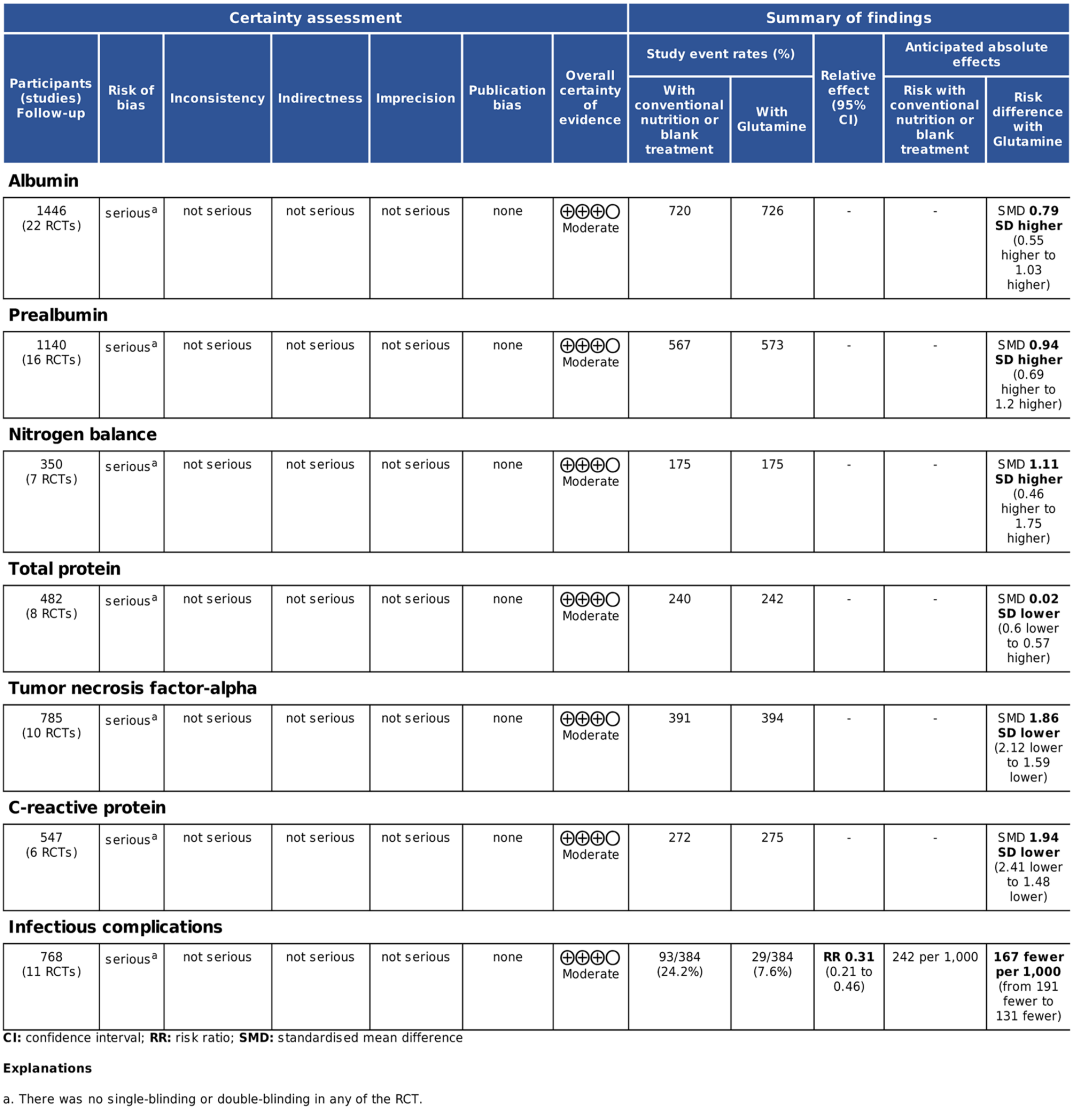
GRADE evaluation for the grades of evidence

Accordingly, we applied the GRADE system to assess the grades of evidence (Fig. 2). The results of the evaluation of these seven indicators showed that the evidence grades for ALB, PA, NB, TP, TNF-α, CRP, and ICs were moderate. The downgrade is a certain degree indicating there was no single-blinding or double-blinding in any of the RCTs, and the the complete absence of blinding which results in a downgrading of the grades of evidence (Fig. 2).

### Evaluation of studies’ quality

**Fig. 3:**
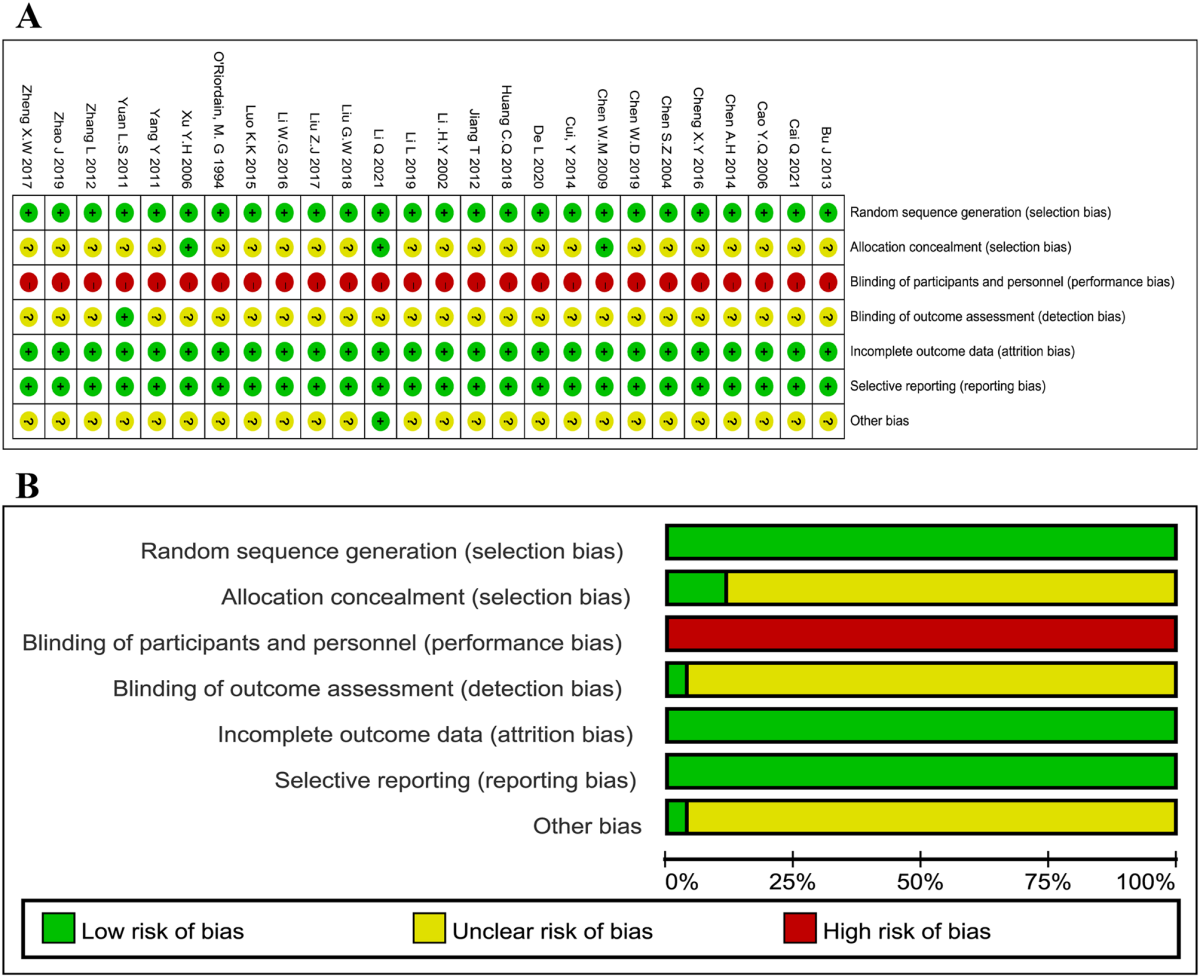
Graph of methodological quality and a summary of the included articles. **A** overview of the risk of bias. **B** graph for the risk of bias

Figure 3 depicts the methodological quality assessments of the 26 articles that were included. As shown in Fig. 3A, in all of the studies that were enrolled, the formation of the randomized sequence was sufficiently recognized. In the vast majority of RCT, the allocation concealment was ambiguous. There was no single-blinding or double-blinding in any of the RCTs. None of the RCTs contained incomplete results or biased reporting. Consequently, the evaluation of performance bias was high risk. The risk of detection bias was uncertain (Fig. 3B). Conclusively, because of the complete absence of blinding, the methodological quality of all chosen studies remained low.

### Meta-analysis findings

#### Impact of glutamine on plasma protein in CRC patients postoperatively

**Fig. 4:**
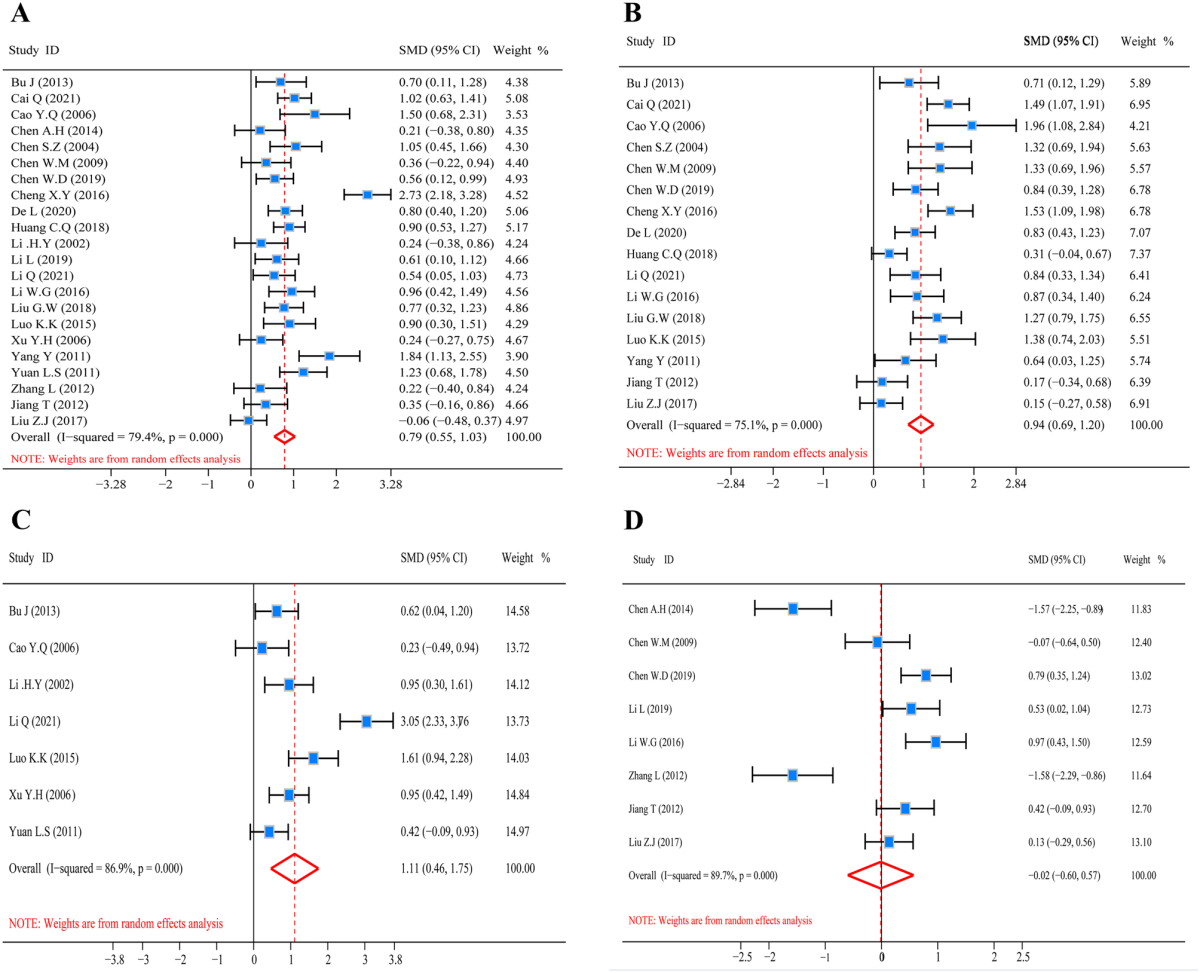
Forest plot of ALB, PA, NB, and TP. **A** Forest plot for ALB. **B** Forest plot for PA. **C** Forest plot for NB. **D** Forest plot for TP. The random-effect model was utilized in all pooled analyses

Figure 4 depicts the pooled analysis of plasma protein markers (ALB, PA, NB, TP). Prior to pooled analysis from these metrics, heterogeneity was investigated. The findings illustrated moderate heterogeneity for ALB (*I*^2^ test = 79.4% and *Q* test *P* = 0.000, Fig. 4A) and for PA (*I*^2^ test = 75.1% and *Q* test *P* = 0.000, Fig. 4B), substantial heterogeneity for NB (*I*^2^ test = 86.9% and *Q* test *P* = 0.000, Fig. 4C) and for TP (*I*^2^ test = 89.7% and *Q* test *P* = 0.000, Fig. 4D) among the selected articles. As a result, for pooled analysis, we utilized the random-effect model. The glutamine cohort had remarkably higher ALB content as opposed to the control cohort, according to the findings (*Z* = 6.35, *P* = 0.000; SMD = 0.79, 95% CI: 0.55 to 1.03; Fig. 4A). The level of PA was also increased in glutamine group (*Z* = 7.30, *P* = 0.000; SMD = 0.94, 95% CI: 0.69 to 1.20; Fig. 4B). Meanwhile, the indicator of NB was significantly increased in glutamine group in contrast with the control group (*Z* = 3.37, *P* = 0.001; SMD = 1.11, 95% CI: 0.46 to 1.75; Fig. 4C). However, the content of TP was no statistical significance (*Z* = 0.05, *P* = 0.959; SMD = − 0.02, 95% CI: − 0.60 to 0.57; Fig. 4D) between the two groups. Those results indicated that glutamine significantly increased the content of ALB and PA, improved the condition of NB for CRC patients after radical operation. But the content of TP in postoperative patients with CRC was not improved in glutamine cohort as opposed to the controls.

#### Impacts of glutamine on inflammatory response of CRC patients postoperatively

**Fig. 5:**
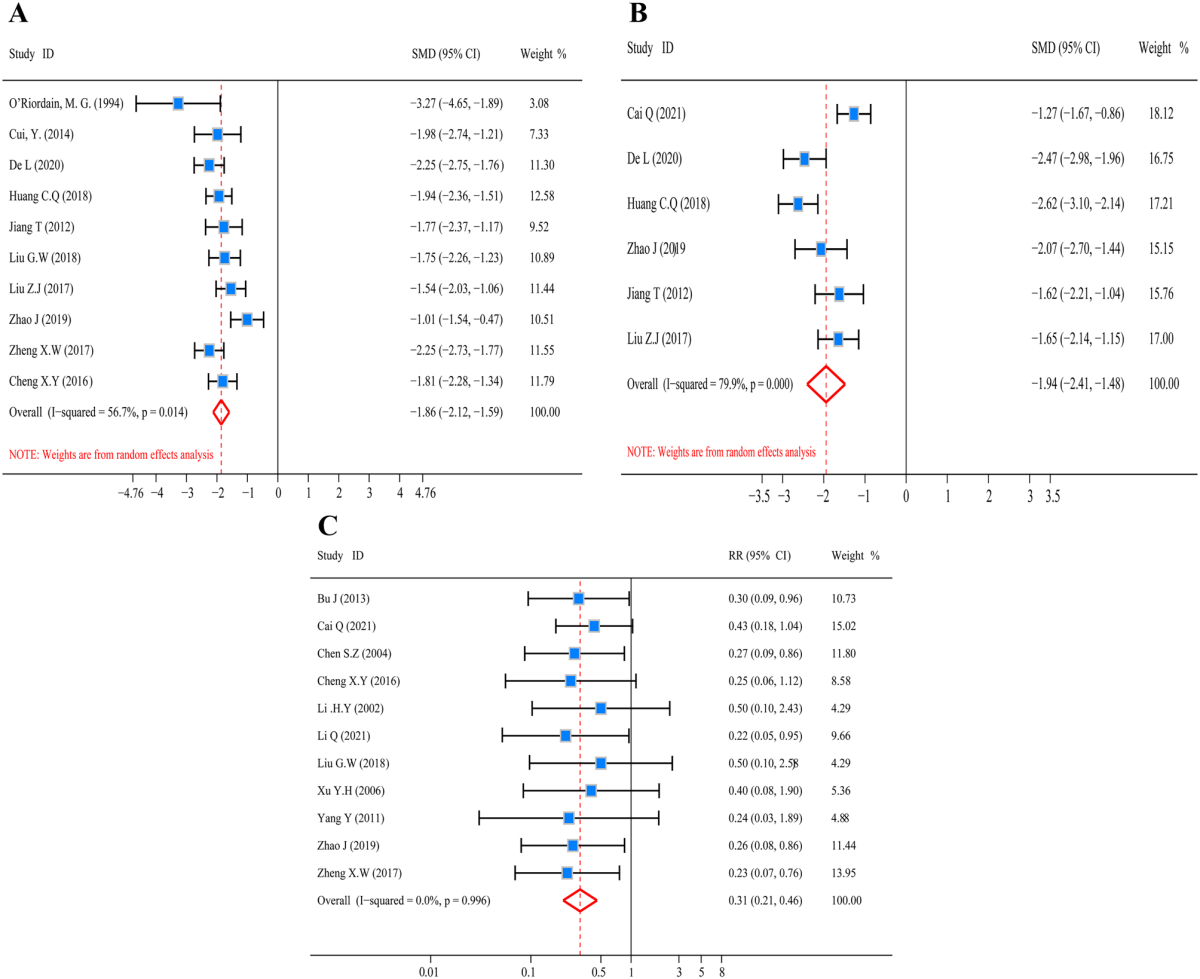
Forest plot for TNF-α, CRP, and ICs. **A** Forest plot of TNF-α and SMD presentation. **B** Forest plot of CRP and SMD presentation. **C** Forest plot of ICs and RR presentation

Before pooled analysis of inflammatory indicators (TNF-α, CRP, and ICs), the articles’ heterogeneity was examined. The findings depicted a slight heterogeneity for TNF-α (*I*^2^ test = 56.7% and *Q* test *P* = 0.014, Fig. 5A), moderate heterogeneity for CRP (*I*^2^ test = 79.9% and *Q* test *P* = 0.000, Fig. 5B) and non-significant heterogeneity for ICs (*I*^2^ test = 0.0% and *Q* test *P* = 0.996, Fig. 5C). Hence, the pooled analysis of CRP and TNF-α was conducted utilizing the random effect model, whereas the pooled analysis of infection-related complications was carried out through the use of the fixed-effect model. The findings recorded from pooled analysis indicated a substantial reduction in the content of TNF-α in the glutamine group in contrast with the controls (*Z* = 13.71, *P* = 0.000; SMD = − 1.86, 95% CI: − 2.21 to − 1.59; Fig. 5A). The level of CRP was decreased significantly in the treatment of glutamine (*Z* = 8.21, *P* = 0.000; SMD = − 1.94, 95% CI: − 2.41 to − 1.48; Fig. 5B). Meanwhile, the incidence of ICs also was decreased significantly in glutamine cohort as opposed to the controls (*Z* = 5.90, *P* = 0.000; RR = 0.31, 95% CI: 0.21 to 0.46; Fig. 5C). The findings illustrated that glutamine significantly extenuated the inflammatory response of postoperative CRC patients.

### Robustness of pooled analysis’ sensitivity

**Fig. 6:**
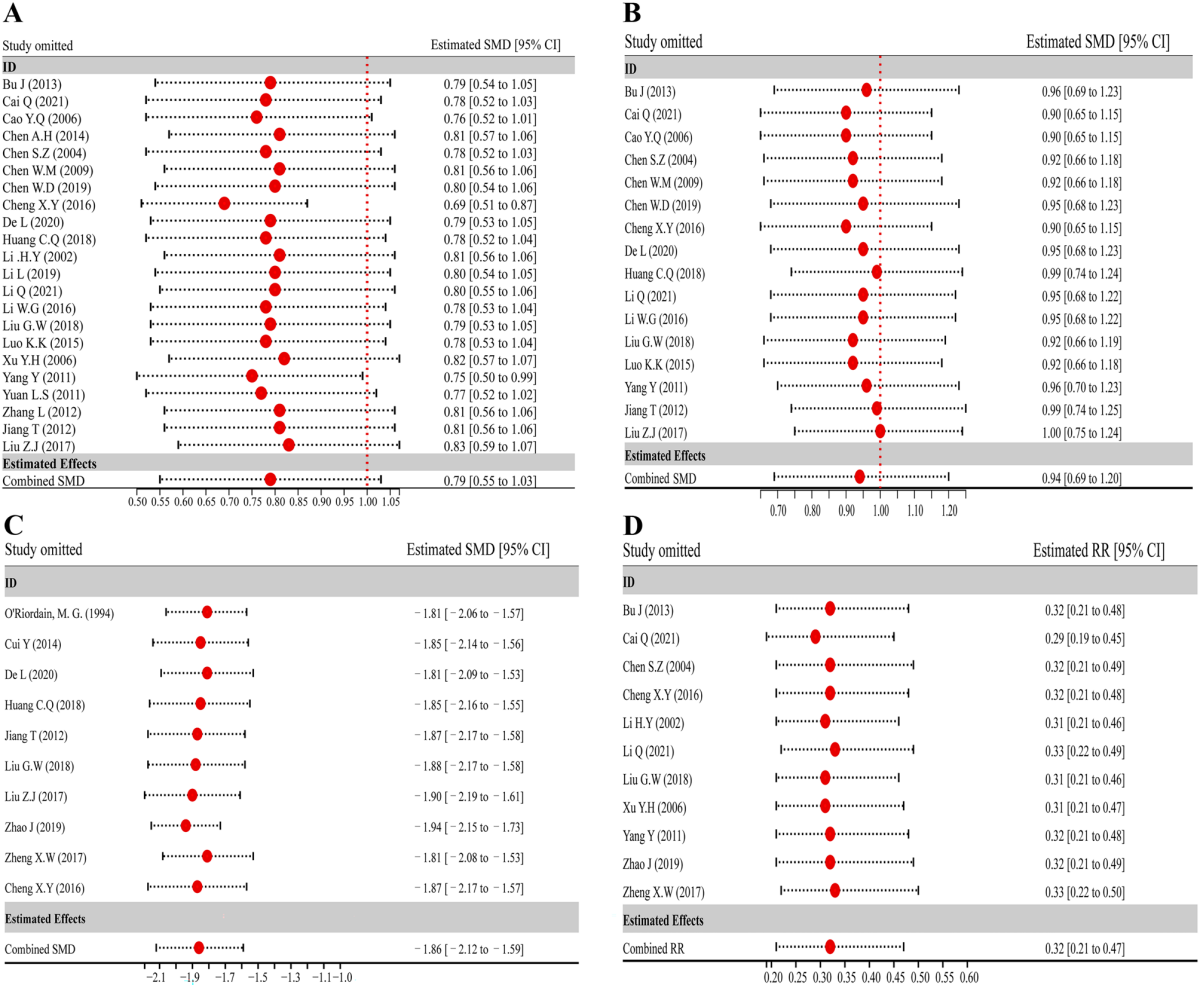
The leave-one-out approach was employed to conduct sensitivity analyses. **A** ALB sensitivity analysis. **B** Analyses of PAs’ sensitivity. **C** TNF-α sensitivity analysis. **D** Analysis of the ICs’ sensitivity

The leave-one-out method was utilized to perform sensitivity analysis in order to determine the robustness of pooled findings (ALB, PA, TNF-α, and ICs) with included RCTs ≥ 10. Sensitivity analysis of ALB results indicated that the study exclusion of Cheng XY[44] could affect the robustness of pooled results, but there were no opposite results (estimated SMD = 0.69, 95% CI: 0.51 to 0.87; Fig. 6A). Meanwhile, the sensitivity analysis of PA (Fig. 6B), TNF-α (Fig. 6C), and ICs (Fig. 6D) revealed the omission of any one of the studies did not significantly affect the robustness and reliability of the pooled analysis. The pooled outcomes were found to have some degree of robustness according to all sensitivity analysis findings.

### Egger’s test for publication bias

**Fig. 7:**
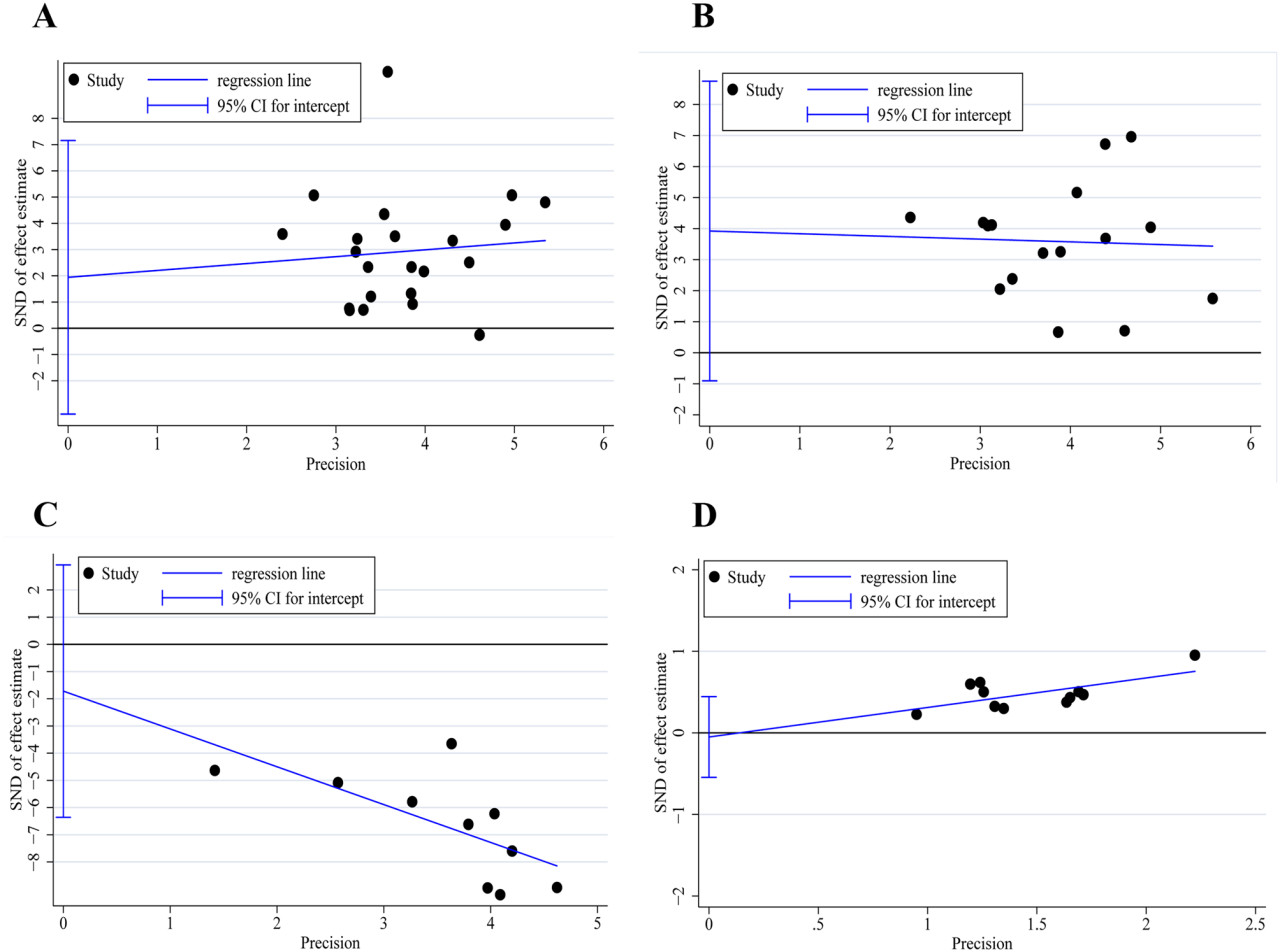
Egger’s funnel plots of ALB, PA, TNF-α, and ICs. **A** Egger’s funnel plot for ALB. **B** Egger’s funnel plot for PA. **C** Egger’s funnel plot for TNF-α. **D** Egger’s funnel plot for ICs

For pooled effects with included articles ≥ 10, we performed Egger’s test to examine probable publication bias. Egger’s test for pooled ALB revealed no small-study effects between included RCTs, the regression funnel plot was symmetrical approximately, and the *P*_ALB_ = 0.446 (Fig. 7A), which indicated no significant significance publication bias in included RCTs. Egger’s test for pooled PA (*P*_PA_ = 0.103, Fig. 7B), TNF-α (*P*_TNF-α_ = 0.418, Fig. 7C), and ICs (*P*_ICs_ = 0.822, Fig. 7D) demonstrated there were no significant publication bias in pooled results. All results of Egger’s test indicated the bias in included RCTs was from performance bias owing to the absence of blinding.

### Possible bias in a contour-enhanced funnel plot

**Fig. 8:**
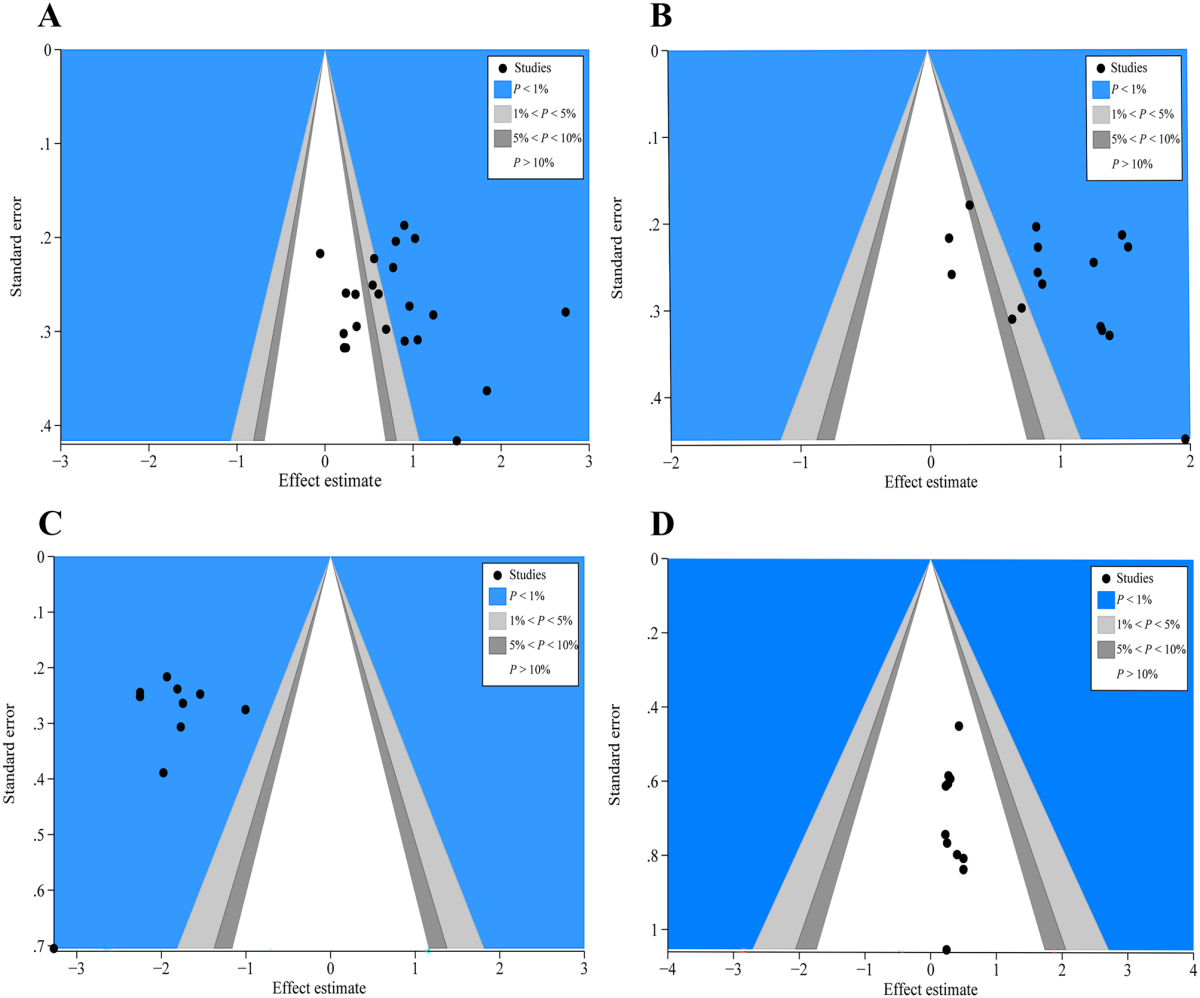
Funnel plots with enhanced contours for ALB, PA, TNF-α, and ICs. **A** ALB’s contour-enhanced funnel plot. **B** PA funnel plot with enhanced contours. **C** TNF-α funnel plot with enhanced contours. **D** IC funnel plot with contour enhancement

To reveal the exact causes of bias, a contour-enhanced funnel plot was utilized, which incorporated standard milestones to funnel plots in terms of statistical significance values (*P* < 0.01, *P* < 0.05,* P* < 0.1, or *P* > 0.1). Contour-enhanced funnel plot of ALB (Fig. 8A), PA (Fig. 8B), and TNF-α (Fig. 8C) indicated many RCTs were missing in terms of high statistical significance (*P* < 0.01), suggesting that the asymmetry of funnel plot was attributable to undiscovered bias instead of publishing bias. Following that, we retraced the primary RCTs and hypothesized that trials having smaller sample sizes, blinding missing or unpublished negative results in several articles might be attributable to those undiscovered biases. On the contrary, the contour-enhanced funnel plot of ICs (Fig. 8D) showed all missing RCTs were within the low statistical significance areas (*P* > 0.1), thereby revealing that the asymmetry of the funnel plot was due to publication bias.

### Meta-regression analysis

**Fig. 9:**
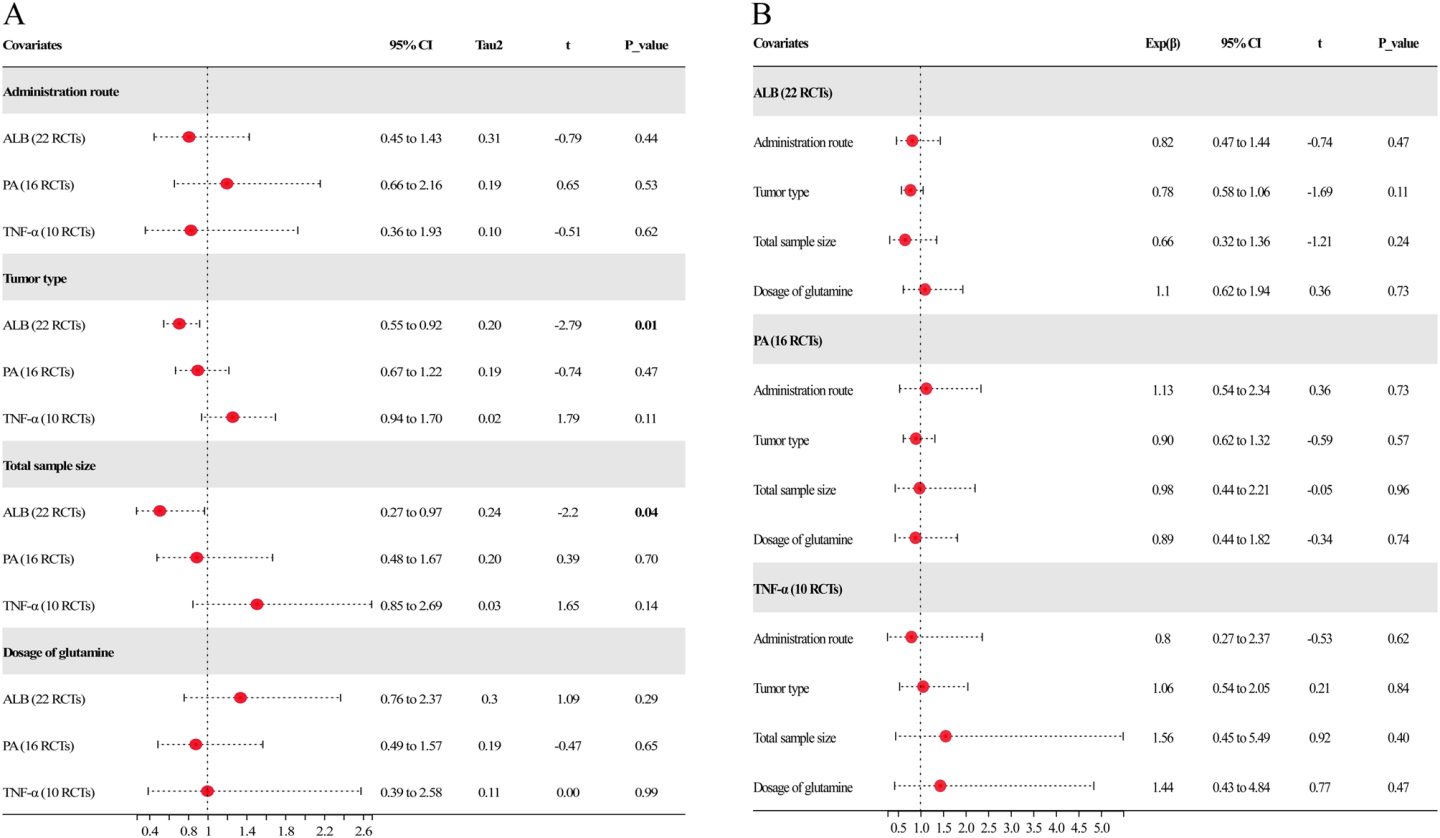
Meta-regression analysis findings. **A** Univariable analysis of meta-regression. **B** Multivariate analysis of meta-regression

There was moderate heterogeneity for the pooled analysis of ALB, PA, and TNF-α. The covariates below were anticipated to have an impact on the estimate of pooled findings and heterogeneity: ① glutamine ingestion route (EN or PN); ② tumor type (colon/rectal/colorectal cancer); ③ size of the sample (≥ 100 or < 100); ④ the dosage of glutamine per day (≥ 0.5 g/kg or < 0.5 g/kg). Succinctly, the univariable analysis illustrated that the covariates of tumor type and sample size (Table 2 and Fig. 9A, P < 0.05) significantly affected the pooled effects of ALB, demonstrating that heterogeneity might be caused by these two covariates. Then, the influence of multi-covariates on the pooled effects was assessed utilizing multivariate meta-regression. The findings highlight that the four mentioned covariates had no influence on the pooled effects of ALB, PA, and TNF-α (*P* > 0.05, Table 2 and Fig. 9B, which suggested interaction effects or collinearity among the four covariates.

**Table 2:** Meta-regression analysis results

Covariates		Univariate analysis				Multivariate analysis		
	Exp(β)	95% CI	Tau^2^	*t*	*P* value	Exp(β)	95% CI	*P* value
**Administration route (PN/EN)**								
ALB (22 RCTs)	0.81	0.45 to 1.43	0.31	− 0.79	0.44	0.82	0.47 to 1.44	0.47
PA (16 RCTs)	1.20	0.66 to 2.16	0.19	0.65	0.53	1.13	0.54 to 2.34	0.73
TNF-α (10 RCTs)	0.83	0.36 to 1.93	0.10	− 0.51	0.62	0.80	0.27 to 2.37	0.62
**Tumor type (colon/rectal/colorectal cancer)**								
ALB (22 RCTs)	0.71	0.55 to 0.92	0.20	− 2.79	**0.01**	0.78	0.58 to 1.06	0.11
PA (16 RCTs)	0.90	0.67 to 1.22	0.19	− 0.74	0.47	0.90	0.62 to 1.32	0.57
TNF-α (10 RCTs)	1.26	0.94 to 1.70	0.02	1.79	0.11	1.06	0.54 to 2.05	0.84
**Total sample size (< 100/ ≥ 100)**								
ALB (22 RCTs)	0.51	0.27 to 0.97	0.24	− 2.20	**0.04**	0.66	0.32 to 1.36	0.24
PA (16 RCTs)	0.89	0.48 to 1.67	0.20	0.39	0.70	0.98	0.44 to 2.21	0.96
TNF-α (10 RCTs)	1.51	0.85 to 2.69	0.03	1.65	0.14	1.56	0.45 to 5.49	0.40
**Dosage of glutamine (≥ 0.5 g/(kg·day) or < 0.5 g/(kg·day)**								
ALB (22 RCTs)	1.34	0.76 to 2.37	0.30	1.09	0.29	1.10	0.62 to 1.94	0.73
PA (16 RCTs)	0.88	0.49 to 1.57	0.19	− 0.47	0.65	0.89	0.44 to 1.82	0.74
TNF-α (10 RCTs)	1.00	0.39 to 2.58	0.11	0.00	0.99	1.44	0.43 to 4.84	0.47

The bolded and underlined findings are significant

## Discussion

Colorectal cancer is a chronic wasting disease and most patients are associated with varying degrees of malnutrition at the time of diagnosis or after surgery [8]. In hypermetabolic and hypercatabolic states, glutamine (an important nutritional substrate for the body’s immune cells and intestinal mucosal cells) is excessively depleted, which can exacerbate intestinal barrier dysfunction, impaired wound healing, and negative nitrogen balance [50]. Although routine early postoperative EN can provide adequate nutritional support for CRC patients, it cannot meet the body’s demand for glutamine in a high metabolic state [32, 48]. What is more, the oxidation of glutamine is a source of nitrogen for other amino acids and proteins, which can play a role in preventing muscle degradation and increasing protein synthesis [14, 15]. In this meta-analysis, we found that nutritional status indicators (ALB, PA, and NB) were improved significantly and inflammatory responses (TNF-α, CRP, and ICs) were reduced significantly by glutamine treatment in CRC patients who underwent curative resection.

CRC radical surgery leads to a high inflammatory burden, which can impair hepatic protein metabolism, leading to malnutrition [51]. A recent propensity-matched study found that glutamine supplementation mitigated the decline in albumin, total protein, and prealbumin levels and reduced ICs in patients who had undergone colorectal cancer surgery, with the difference being statistically significant [52]. In addition, Yang et al.’s meta-analysis for CRC surgery revealed glutamine significantly decreased the duration of hospitalization, postoperative complications that containing the surgical site infection and anastomotic leakage [53]. These results are broadly similar to our findings, but we focused on comparing the effects of glutamine supplementation on plasma proteins in postoperative CRC patients and included more studies [52, 53]. Moreover, previous cohort study with a large sample size (*n* = 1950) has indicated glutamine administration exhibited a positive dosage-dependent influence on restoring the levels of serum ALB in gastrectomy patients, demonstrating that glutamine enhanced nutritional status following surgical operation and reduced the incidence of stress-related inflammation [50]. And a prospective study showed that the fat-free mass, serum ALB, quality of life, and postoperative outcomes were significantly improved by glutamine supplementation [54]. Klek et al. found that although standard EN was similar to immunized EN in terms of infectious complications and protein synthesis, it was more costly than immunized EN after gastrointestinal surgery [55]. Therefore, glutamine-enhanced EN in the perioperative should be a superior option for postoperative patients with CRC.

ALB reflects the malnutritional state of the host and triggers tumor progression and malignancy [56]. Plasma albumin levels are disturbed by a variety of physical interactions, such as hepatic albumin synthesis, vascular permeability, and interstitial volume. The inflammatory effect produced by surgical trauma leads to an increase in intracapillary permeability, which allows plasma proteins to escape into the interstitium. And this returns when the inflammation subsides [56, 57]. This may be the reason for the decrease in serum ALB after CRC surgery.

The mechanism by which glutamine elevates serum ALB and TP in postoperative CRC patients may be as follows. Liver: During severe illness, the liver is the main consumer of glutamine[58]. Glutamine deprivation leads to an impaired ability of liver cells to synthesize proteins. What is more, following glutamine ingestion, hepatocytes is swelling and enhancing hydration [59]. Then, glycogen and fatty acid synthesis increases and P38 mitogen-activated protein kinase (P38 MAPK) signaling-mediated protein hydrolysis decreases [58, 60]. Gut: Glutamine can significantly promote the recovery of intestinal function, enhance the intestinal mucosal barrier function, promote the repair of intestinal mucosa and ulcers, enhance intestinal absorption function, enhance immune function and improve intestinal permeability [61, 62]. These can promote protein absorption and reduce protein leakage.

Inadditionly, the findings recorded from pooled analysis depicted that glutamine reduced the level of TNF-α and CRP in postoperative patients of CRC when compared with the conventional nutrition support group. Increasing evidence substantiated that glutamine reduced pro-inflammatory mediators secretion in the mucosa of the human intestine and decreased infectious morbidity [42, 63]. A clinical trial has reported glutamine leads to an increase in the total lymphocyte count, CD8 +, CD4 +, complement IgA, IgG, C3 in immunocompromised patients and lowered the content of CRP as well as the risks of incision infections[64]. Results from a double-blind RCTs indicated that glutamine administration significantly down-regulated the pro-inflammatory mRNA expression of interleukin-1β, CD36, toll-like receptor 4, TNF-α, cyclooxygenase-2, matrix metalloproteinase-9, activator protein 1, and nuclear factor kappa B, among cancer patients following abdominal radiotherapy[63]. Furthermore, the content of inflammation-related cytokines (interleukin-6 and CRP) was also significantly reduced in postoperative cancer patients [65]. Consequently, we conclude that glutamine administration might ameliorate ampliative inflammatory responses following radical surgical procedures in patients with CRC.

However, there are some limitations in this article. Firstly, none of the 26 selected RCTs had single or double-blinding designs, thereby increasing detection bias risk and de-escalating the grades of pooled evidence. Secondly, the outcomes of the contour-enhanced funnel plot revealed that trials having smaller sample sizes, missing or unreported negative effects, and blinding might all explain the undiscovered bias. Thirdly, meta-regression via the univariate analysis revealed that tumor type and sample sizes in original RCTs were the potential covariates attributable to the heterogeneity and de-escalating the validity of pooled analysis. Despite we tried to maintain the homogeneity of included studies through strict inclusion and exclusion criteria, the heterogeneity is inevitable among all the studies included in the meta-analysis. The protocol of “PICOS” of each study is different, which leads to the existence of clinical heterogeneity. In addition, the original studies were carried out with different RCT methods, for example, some studies were single- or double-blind and some were open-label design, with large or small sample size, et al., which leads to the methodological heterogeneity. Therefore, we attributed the significant heterogeneity between included studies to the clinical heterogeneity and methodological heterogeneity.

## Conclusion

This meta-analysis comprising 1678 CRC cases from 26 RCTs confirmed that glutamine supplementation substantially improved nutritional status and reduced inflammatory reactions in CRC patients following surgical interventions. Before affirming the findings of this research, it is important to keep in mind the drawbacks of the methodology. The presence of multiple disciplinary teams (MDTs) in the preoperative or postoperative phases of CRC care is generally acknowledged, as is the requirement for long-term pharmacotherapy. To counterbalance the risk–benefit profile of glutamine in the therapy of CRC surgical intervention, further RCTs with a larger scale and multi-dimensional effectiveness and nutritional status evaluation are needed.

### Supplementary Information

Below is the link to the electronic supplementary material.

Supplementary file1 (DOCX 32 KB)

Supplementary file2 (DOCX 13 KB)

## Data Availability

All data relevant to the study are included in the article or uploaded as supplementary information.
