# Supplementary material for: Effects of glutamine on plasma protein and inflammation in postoperative patients with colorectal cancer: a meta-analysis of randomized controlled trials
Source: Int J Colorectal Dis. 2023 Aug 11;38(1):212. doi: 10.1007/s00384-023-04504-8 (PMC10421765; doi:10.1007/s00384-023-04504-8)
Supplement: Supplementary file 2 — Supplementary file2 (DOCX 13 KB) [file 384_2023_4504_MOESM2_ESM.docx]

Taking the PubMed database as an example, the detailed search syntax are as follows:

#1 Colorectal cancer[MeSH Terms]

#2 Rectal cancer[MeSH Terms]

#3 Colon cancer[MeSH Terms]

#4 #1 OR #2 OR #3

#5 Tumor af.

#6 Carcinoma af.

#7 Neoplasm af.

#8 #5 OR #6 OR #7

#9 #4 OR #8

#10 Glutamine[MeSH Terms]

#11 Glutamine af.

#12 Gln af.

#13 Nutrition af.

#14 immune-nutrition af.

#15 #10 OR #11 OR #12 OR #13 OR #14

#16 #9 AND #15

#17 Random af.

#18 Randomized af.

#19 Trials af.

#20 RCTs af.

#21 #17 OR #18 OR #19 OR #20

#22 #16 AND #21

**Search:** ((((((Colorectal cancer[MeSH Terms])) OR ((Rectal cancer[MeSH Terms]))) OR ((Colon cancer[MeSH Terms]))) OR (Colorectal cancer)) AND (Glutamine)) AND (((random) OR (trials)) OR (RCTs))

("colorectal neoplasms"[MeSH Terms] OR "rectal neoplasms"[MeSH Terms] OR "colonic neoplasms"[MeSH Terms] OR ("colorectal neoplasms"[MeSH Terms] OR ("colorectal"[All Fields] AND "neoplasms"[All Fields]) OR "colorectal neoplasms"[All Fields] OR ("colorectal"[All Fields] AND "cancer"[All Fields]) OR "colorectal cancer"[All Fields])) AND ("glutamin"[All Fields] OR "glutamine"[MeSH Terms] OR "glutamine"[All Fields] OR "glutamine s"[All Fields] OR "glutamines"[All Fields]) AND ("random allocation"[MeSH Terms] OR ("random"[All Fields] AND "allocation"[All Fields]) OR "random allocation"[All Fields] OR "random"[All Fields] OR "randomization"[All Fields] OR "randomized"[All Fields] OR "randomisation"[All Fields] OR "randomisations"[All Fields] OR "randomise"[All Fields] OR "randomised"[All Fields] OR "randomising"[All Fields] OR "randomizations"[All Fields] OR "randomize"[All Fields] OR "randomizes"[All Fields] OR "randomizing"[All Fields] OR "randomness"[All Fields] OR "randoms"[All Fields] OR ("clinical trials as topic"[MeSH Terms] OR ("clinical"[All Fields] AND "trials"[All Fields] AND "topic"[All Fields]) OR "clinical trials as topic"[All Fields] OR "trial"[All Fields] OR "trial s"[All Fields] OR "trialed"[All Fields] OR "trialing"[All Fields] OR "trials"[All Fields]) OR "RCTs"[All Fields])
